# A Strategy for Tuning the Structure, Morphology, and Magnetic Properties of MnFe_2_O_4_/SiO_2_ Ceramic Nanocomposites via Mono-, Di-, and Trivalent Metal Ion Doping and Annealing

**DOI:** 10.3390/nano13142129

**Published:** 2023-07-22

**Authors:** Thomas Dippong, Erika Andrea Levei, Ioan Petean, Iosif Grigore Deac, Oana Cadar

**Affiliations:** 1 Faculty of Science, Technical University of Cluj-Napoca, 76 Victoriei Street, 430122 Baia Mare, Romania; 2 INCDO-INOE 2000, Research Institute for Analytical Instrumentation, 67 Donath Street, 400293 Cluj-Napoca, Romania; 3 Faculty of Chemistry and Chemical Engineering, Babes-Bolyai University, 11 Arany Janos Street, 400028 Cluj-Napoca, Romania; 4 Faculty of Physics, Babes-Bolyai University, 1 Kogalniceanu Street, 400084 Cluj-Napoca, Romania

**Keywords:** manganese ferrite, silica matrix, doping, annealing, magnetic behavior

## Abstract

This work presents the effect of monovalent (Ag^+^, Na^+^), divalent (Ca^2+^, Cd^2+^), and trivalent (La^3+^) metal ion doping and annealing temperature (500, 800, and 1200 °C) on the structure, morphology, and magnetic properties of MnFe_2_O_4_/SiO_2_ ceramic nanocomposites synthesized via sol–gel method. Fourier-transform infrared spectroscopy confirms the embedding of undoped and doped MnFe_2_O_4_ nanoparticles in the SiO_2_ matrix at all annealing temperatures. In all cases, the X-ray diffraction (XRD) confirms the formation of MnFe_2_O_4_. In the case of undoped, di-, and trivalent metal-ion-doped gels annealed at 1200 °C, three crystalline phases (cristobalite, quartz, and tridymite) belonging to the SiO_2_ matrix are observed. Doping with mono- and trivalent ions enhances the nanocomposite’s structure by forming single-phase MnFe_2_O_4_ at low annealing temperatures (500 and 800 °C), while doping with divalent ions and high annealing temperature (1200 °C) results in additional crystalline phases. Atomic force microscopy (AFM) reveals spherical ferrite particles coated by an amorphous layer. The AFM images showed spherical particles formed due to the thermal treatment. The structural parameters calculated by XRD (crystallite size, crystallinity, lattice constant, unit cell volume, hopping length, density, and porosity) and AFM (particle size, powder surface area, and thickness of coating layer), as well as the magnetic parameters (saturation magnetization, remanent magnetization, coercivity, and anisotropy constant), are contingent on the doping ion and annealing temperature. By doping, the saturation magnetization and magnetocrystalline anisotropy decrease for gels annealed at 800 °C, but increase for gels annealed at 1200 °C, while the remanent magnetization and coercivity decrease by doping at both annealing temperatures (800 and 1200 °C).

## 1. Introduction

Nanoparticles display enhanced properties relative to microparticles and bulk materials, allowing their use in various applications [1]. Spinel ferrite (MFe_2_O_4_, M=Mn, Ni, Co, Cu, etc.) nanoparticles are developing as a family of versatile materials with controllable particle size and shape, tunable dielectric, catalytic, and magnetic properties, as well as easy and convenient synthesis processes [1,2,3,4,5,6]. Of these, manganese ferrite, MnFe_2_O_4_, displays a face-centered cubic structure with two types of cation lattice sites: tetrahedral (A) formed by four O^2-^ ions, and octahedral (B) sites composed of six O^2–^ ions. The percentage of Fe^3+^ ions occupying the A sites dictates the inversion degree. Thus, in normal spinel structure the A sites are occupied by Fe^3+^ ions, while in inverse spinel structure the A sites are occupied by Mn^2+^ ions [1]. The inversion degree highly influences the magnetic properties of MnFe_2_O_4_ nanoparticles. MnFe_2_O_4_ has attracted significant interest due to its controllable grain size, superparamagnetic nature, low coercivity (*H_C_*), high magnetic permeability, moderate saturation magnetization (*M_S_*), good chemical stability, high catalytic performance, capacity to be guided by an external magnetic field, surface tailoring possibility, good biocompatibility, and high crystal symmetry [1,5,7]. MnFe_2_O_4_ is also a non-toxic, non-corrosive, environmentally friendly, high thermal, and shock-resistant material often used for application in medicine, electronics, as well as in the paint and coating industry [1,2,3,4,5,8].

Doping with various cations enhances the ferrites’ magnetic, optical, and electrical properties. The doped spinel ferrites have various benefits, i.e., they are less expensive, easy to produce, have good stability, and have different magnetic properties compared to undoped ferrites [9]. Cation distribution is significantly affected by the doping ion radius, charge, lattice energy, and crystal field stabilization energy in A and B sites [10]. In recent years, a large interest has been granted to doped ferrites due to their numerous technical applications, including magnetically controlled anticancer medication delivery, color imaging, and gas-sensitive and catalytic materials [9,10,11]. Metal ion doping generates oxygen vacancies and reactive oxygen species that enhance the catalytic performance [9]. In Zn^2+^-doped MnFe_2_O_4_, Zn has a strong tendency to occupy A sites enhancing the magnetic properties [10,12]. Previous studies reported that the Mn–Zn ferrites decompose by annealing, leading to impure phases and, consequently, the decrease in magnetic and dielectric properties [10,13]. The high stability and outstanding electrical and thermal conductivity of Ag make it a dopant that improves the catalytic activity of MnFe_2_O_4_, allowing the degradation of refractory organic pollutants [6]. Ag–Mn_2_Fe_2_O_4_ composites also display superparamagnetic and remarkably antibacterial activity [14]. A previous study on Ca–MnFe_2_O_4_ nanoparticles coated with citrate obtained by the sol-gel method revealed that high Ca content improves their capacity to be used as a hyperthermia agent without compromising their cytocompatibility or cellular internalization [15]. Recently, the structural tuning of MnFe_2_O_4_ by doping with rare earth ions has drawn attention as a novel technique to enhance its physical characteristics. Of these, the non-magnetic La^3+^ ion stands out due to its larger ionic radius compared to that of Fe^3+^ and Mn^2+^ ions that change the structural and magnetic properties of MnFe_2_O_4_ by the higher electron–hole pair recombination in the ferrites, supporting the shift of the electronic states [7,16]. Moreover, La^3+^ doping promotes a higher production of oxygen vacancies and photocatalytic degradation [7,16].

Given the diversity of experimental techniques (e.g., sol–gel, hydrothermal, thermal decomposition, colloid emulsion, and laser pyrolysis) used to obtain MnFe_2_O_4_ ferrites, the sol–gel route offers a flexible approach due to its low cost, low reaction temperature, simplicity, and good control of chemical composition, structural, physical–chemical, and magnetic properties [2,3,4,5]. The prolonged exposure to synthesis conditions, as well as the time of thermal processing, was found to influence the structure of the nanomaterials [17]. Solvo/hydrothermal synthesis is an environmentally friendly approach to producing small and uniformly distributed nanostructures. It also allows the easy doping and coating of the particles to generate composite materials [18]. Microwave-assisted solvothermal approach allows the fine control of process parameters, high productivity, exceptional phase purity, good reproducibility, and short reaction times concomitantly obtaining small particles with uniform particle morphology and high crystalline particles [19]. Baublytė et al. [20] showed a correlation between precursor concentration, particle size, and crystallinity.

The embedding of MnFe_2_O_4_ in mesoporous SiO_2_ plays an important role in enhancing the stability in water, improving biocompatibility, and diminishing the degradation of MnFe_2_O_4_ nanoparticles. The SiO_2_ coating also prevents agglomeration by controlling the dipolar attraction between the magnetic nanoparticles. Moreover, the silanol groups from the surface of mesoporous SiO_2_ promote the binding of biomolecules, directing targeted ligands and drug loading on the nanocarrier surface [2,3,4,5]. Our previously reported sol–gel synthesis method allows the obtaining of homogeneous pure or mixed ferrite nanoparticles and their incorporation in inorganic or organic matrices, requires reduced time and energy and has a short gelation time. The obtaining of MnFe_2_O_4_ embedded in the SiO_2_ matrix sol–gel method consist in the mixing of reactants with tetraethylorthosilicate (TEOS) and the formation of strong networks with moderate reactivity that permit the incorporation of various inorganic and organic molecules [2,3,4,5]. The simple variation in synthesis conditions such as pH, time and annealing temperature allows a high degree of control over the nucleation and particle growth [1,2,3,4,5,6]. The easily controllable magnetic, electrical, dielectric, and optical properties of MnFe_2_O_4_/SiO_2_ nanocomposites recommend their use in various technological and scientific systems, such as magnetic devices, catalysis, and sensors [3,5,21,22]. The MnFe_2_O_4_/SiO_2_ nanocomposites display remarkable electrical and magnetic properties, high chemical and thermal stability, improved microwave absorption performance owing to the strong eddy current loss, excellent attenuation characteristic, better impedance matching, and multiple Debye relaxation processes [23]. The magnetic MnFe_2_O_4_/SiO_2_ nanocomposites are widely studied due to their potential applications in different areas such as electronic, microwave, and communication devices, information storage systems, ferrofluid technology, gas sensors, magnetocaloric refrigeration, and for photocatalytic activity [1,24,25,26,27]. Moreover, the MnFe_2_O_4_ and SiO_2_ integrated into a single entity (nanocomposites particle) is of particular interest in magnetic fluid hyperthermia due to MnFe_2_O_4_ superior magnetization and biocompatibility of SiO_2_ [28] and drug delivery applications by providing the advantages of mesoporous silica surface (e.g., drug loading and surface functionalization) and the magnetic nature of MnFe_2_O_4_ nanoparticles (e.g., magnetic controllability and targeted drug delivery) [29]. 

The architecture adopted by ferrites depends on the metal ion(s) size, charge and concentration, crystal field effects and electrostatic contribution to the lattice energy, while the particle size increase and the volume-to-surface ratio decreases with annealing temperature. Moreover, due to its high degree of magnetization compared to other nanoferrites, MnFe_2_O_4_ has become important for various biomedical applications. Additionally, tailoring MnFe_2_O_4_ by doping with various ions could enhance its magnetic, optical, and electrical properties. Thus, producing homogenous doped MnFe_2_O_4_ nanoparticles with tailored magnetic properties and crystalline structures is challenging, but it is important to discover novel approaches to increase their potential for existing and new conceivable applications. In this regard, this study was conducted to assess the changes in structure, morphology, surface, and magnetic properties of MnFe_2_O_4_ doped with monovalent (Ag^+^, Ag_0.1_Mn_0.95_Fe_2_O_4_; Na^+^, Na_0.1_Mn_0.95_Fe_2_O_4_), divalent (Ca^2+^, Ca_0.1_Mn_0.9_Fe_2_O_4_; Cd^2+^, Cd_0.1_Mn_0.9_Fe_2_O_4_), and trivalent (La^3+^, La_0.1_MnFe_1.9_O_4_) metal ions embedded in a SiO_2_ matrix synthesized through a modified sol–gel method, followed by annealing at 500, 800, and 1200 °C. 

## 2. Materials and Methods

### 2.1. Reagents

All chemicals were used as received without further purification and purchased from different commercial sources as follows: manganese nitrate tetrahydrate (Mn(NO_3_)_2_∙4H_2_O, Merck, Darmstadt, Germany), ferric nitrate nonahydrate (Fe(NO_3_)_3_∙9H_2_O, 98%, Merck, Darmstadt, Germany), silver nitrate (AgNO_3_, 99%, Carlo Erba, Milan, Italy), sodium nitrate (NaNO_3_, 99%, Merck, Darmstadt, Germany), calcium nitrate tetrahydrate (Ca(NO_3_)_2_∙4H_2_O, 99%, Carlo Erba, Milan, Italy), cadmium nitrate tetrahydrate (Cd(NO_3_)_2_∙4H_2_O, 99%, Carlo Erba, Milan, Italy), lanthanum nitrate hexahydrate (La(NO_3_)_3_∙6H_2_O, 98%, Carlo Erba, Milan, Italy) 1,3 propanediol (1,3–PD, 99%, Merck, Darmstadt, Germany), TEOS (99%, Merck), and ethanol (96%, Merck, Darmstadt, Germany). 

### 2.2. Synthesis

MnFe_2_O_4_, Ag_0.1_Mn_0.95_Fe_2_O_4_, Na_0.1_Mn_0.95_Fe_2_O_4_, Ca_0.1_Mn_0.9_Fe_2_O_4_, Cd_0.1_Mn_0.9_Fe_2_O_4_, and La_0.1_MnFe_1.9_O_4_ embedded in SiO_2_ gels containing 50 wt.% ferrite and 50 wt.% SiO_2_ were prepared through a modified sol–gel route using different M/Co/Fe (M = Ag, Na, Ca, Cd, La) molar ratios, namely 0/1/2 (MnFe_2_O_4_), 1/9.5/20 (Ag_0.1_Mn_0.95_Fe_2_O_4_, Na_0.1_Co_0.95_Fe_2_O_4_), 1/9/20 (Cd_0.1_Co_0.9_Fe_2_O_4_, Ca_0.1_Co_0.9_Fe_2_O_4_), and 1/10/19 (La_0.1_CoFe_1.9_O_4_). The key advantages of the sol-gel method are versatility, simplicity, effectiveness, achievement of high purity products, narrow particle size distribution, and uniform nanostructure at low temperatures. The main disadvantages refer to the presence of amorphous phases at low annealing temperatures and secondary crystalline phases at high annealing temperatures, respectively [22]. Briefly, the sol–gel method used here involves the following steps: the reactants (metal nitrates and polyols) are mixed with TEOS at ambient temperature, the sol is exposed to ambient temperature until the gelation of the SiO_2_ network, followed by the thermal-assisted formation of carboxylate precursors and their decomposition to a multicomponent system (mixed oxide). Generally, to obtain spinel ferrites via the sol–gel method, nitrate salts are preferred as precursors, as they are a convenient source of aqueous metal ions and act as low-temperature oxidizing agents for the synthesis [22,30]. Accordingly, the initial sols were prepared by mixing the metal nitrates with 1,3–PD, TEOS and ethanol using a NO_3_^–^/1,3–PD/TEOS molar ratio of 1/1/1. The resulting sols were vigorously stirred over a 1 h and kept at ambient air until gelation occurred. The obtained gels consisting of a homogenous mixture of TEOS, 1,3–PD and metal nitrates were ground thoroughly using an agate mortar pestle and heated at 40 °C for 5 h and 200 °C for 5 h, respectively, in an UFE 400 universal oven (Memmert, Schwabach, Germany). Finally, the powder samples were annealed at different temperatures (500, 800 and 1200 °C) for 5 h at 10 °C/min using a LT9 (Nabertherm, Lilienthal, Germany) muffle furnace, at ambient temperature.

### 2.3. Characterization

X-ray diffraction patterns were recorded on a D8 Advance (Bruker, Karlsruhe, Germany) diffractometer equipped with an X-ray tube (CuKα radiation, λ = 1.54060 Å, 40 kV and 35 mA) and 1–dimensional LynxEye detector; data collection was carried out in the 2θ range of 15–80°, with a step size of 0.015° and counting time of 1s/step. The Fourier-transform infrared (FT–IR) spectra in the range of 400–4000 cm^−1^ were recorded in transmittance mode with a resolution of 2 cm^−1^ and 8 scans on KBr pellets containing 1% sample using a Perkin–Elmer Spectrum BX II (Perkin Elmer, Waltham, MA, USA) spectrometer equipped with DTGS detectors. For atomic force microscopy (AFM), the thermally treated powders were dispersed in ultrapure water by stirring to enable the finest particles to release. Bigger particles sediment on the bottom of the vials, while the finest ferrite particles remained dispersed due to Brownian motion. They were transferred onto a glass slide via vertical adsorption, and the formed thin films were dried at room temperature and investigated using a JSPM 4210 (JEOL, Tokyo, Japan) microscope. The AFM was operated in alternative current mode with NSC 15 (Mikromasch, Sofia, Bulgaria) cantilevers with a nominal force constant of 40 N/m frequency of 325 kHz. At least three macroscopic areas of 1 µm^2^ of the thin films were scanned to obtain the topographic images. The thin ferrite films’ height, surface roughness (Rq), particle diameter, and surface area were obtained by analyzing the images with WinSPM System Data Processing Version 2.0 by JEOL. The magnetic measurements were carried out at room temperature using a cryogen free vibrating-sample magnetometer (VSM), CFSM—12 T (Cryogenic Ltd., London, UK). The hysteresis loops were recorded with a maximum field of 2 T, while the magnetization was measured in a high magnetic field up to 7 T. The powder samples were embedded into epoxy resin to avoid the motion of particles.

## 3. Results and Discussion

### 3.1. FT–IR Analysis

**Figure 1:**
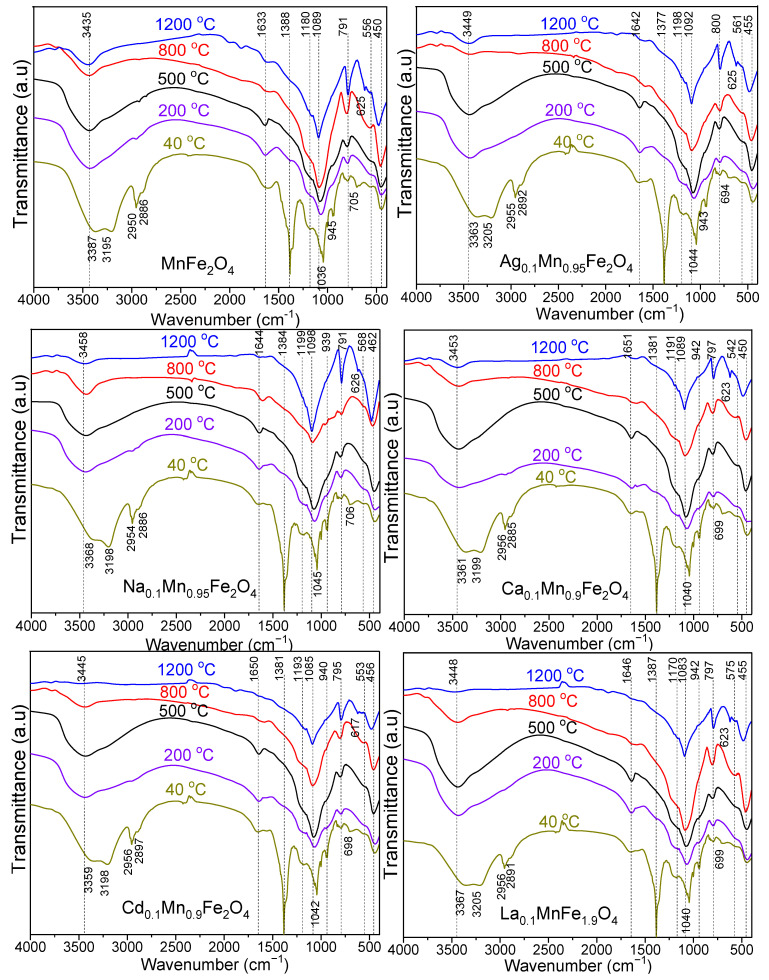
FT–IR spectra of MnFe_2_O_4_, Ag_0.1_Mn_0.95_Fe_2_O_4_, Na_0.1_Mn_0.95_Fe_2_O_4_, Ca_0.1_Mn_0.9_Fe_2_O_4_, Cd_0.1_Mn_0.9_Fe_2_O_4_, and La_0.1_MnFe_1.9_O_4_ gels thermally treated at 40, 200, 500, 800, and 1200 °C.

The functional groups, molecular geometry and inter-molecular interactions remarked in the FT–IR spectra of undoped and doped MnFe_2_O_4_ thermally treated at 40, 200, 500, 800, and 1200 °C are presented in Figure 1.

In all cases, the FT–IR spectra show the representative bands of the SiO_2_ matrix, namely: O–H bond vibration in the Si–OH group (3435–3458 cm^−1^), H–O–H bond bending vibration (1633–1651 cm^−1^), Si–O–Si bonds stretching vibration (1083–1098 cm^−1^), Si–O chains vibration in SiO_4_ tetrahedron (791–800 cm^−1^), Si–O bond vibration (450–462 cm^−1^) and Si–O–Si cyclic structures vibration (542–575 cm^−1^) [2,3,4,5,16]. The deformation vibration of Si–OH resulted during the hydrolysis of –Si(OCH_2_CH_3_)_4_ groups of TEOS in gels dried at 40 °C shown by the shoulder at 939–945 cm^−1^ disappears at higher temperatures. The water is present on the surface and in the volume of SiO_2_ particles in physically and chemically (bounded to the surface and molecularly dispersed) bound forms [31,32]. The physiosorbed water does not interact strongly with the particle’s surface and it can be easily removed at low temperatures, whereas the chemisorbed water is removed at higher temperatures [32]. Removing water from most metal oxide nanoparticles may be incomplete regardless of the used temperature and lead to coarsening and phase transformation [32].

For gels dried at 40 °C, the intense band at 1633 cm^−1^ is attributed to –O–H bond vibrations in the diol, physically and chemically bound water molecules [2,3,4,5,16,32], while for gels thermally treated at 200 °C this band shifts to 1642 cm^−1^ and is attributed to the C=O bond vibration in the carboxylate precursors and the chemically absorbed water molecules [31]. Increasing the thermal treatment temperature, this band progressively decreases following the decomposition of carboxylate precursors and loss of chemically absorbed water, until it disappears at 1200 °C [16]. The presence of this band at high temperatures could be explained by the high hygroscopicity of the nanoparticles and the presence of polar hydroxyl groups, both on the silica surface and in its volume [32]. The dissociation of chemically adsorbed water on the particle surface with the formation of hydroxyl groups stabilizes the surface, reduces the water mobility, and increases the nanoparticle’s stability [32].

In the case of gels dried at 40 °C, the intense band at 1633 cm^−1^ is attributed to the overlapping of C–O and H–O deformation vibrations [2,3,4,5,16]. This band shifts to 1642 cm^−1^ in the case of gels thermally treated at 200 °C and is attributed to the C=O bond vibration in the carboxylate precursors; by further increasing the thermal treatment temperature, it progressively decreases till it disappears at 1200 °C [33]. In gels thermally treated at 40 °C, the broad bands at 3359–3387 cm^−1^ and 3195–3205 cm^−1^ are attributed to O–H stretching in precursors and to intermolecular hydrogen bonds [16,34]. The intense band around 1377–1388 cm^−1^ present only in gels dried at 40 °C is characteristic of N–O bond’s asymmetric vibration in nitrates. The absence of this band at high temperatures suggests that the reaction between nitrates and 1,3–PD with the formation of metal–carboxylate precursors has already occurred [16]. The asymmetric and symmetric bands at 2950–2956 cm^−1^ and 2885–2897 cm^−1^ present only in gels at 40 °C, are characteristic of C–H bond vibration in the methylene groups of 1,3–PD and carboxylates precursors and disappear at higher temperatures when the precursors decompose [16,34].

The vibration of M–O bonds in A sites is indicated by the band at 542–575 cm^−1^ and in B sites by the band at 450–462 cm^−1^ [16]. The bands at 617–626 cm^–1^ (1200 °C) and 694–706 cm^–1^ (200 °C) are attributed to O–Fe–O and Fe–OH bond vibration [33,34]. The band shift is ascribed to the modification in M−O bond length at the A and B sites due to the introduction of large size rare earth La^3+^ ion [7].

### 3.2. Structural Analysis

**Figure 2:**
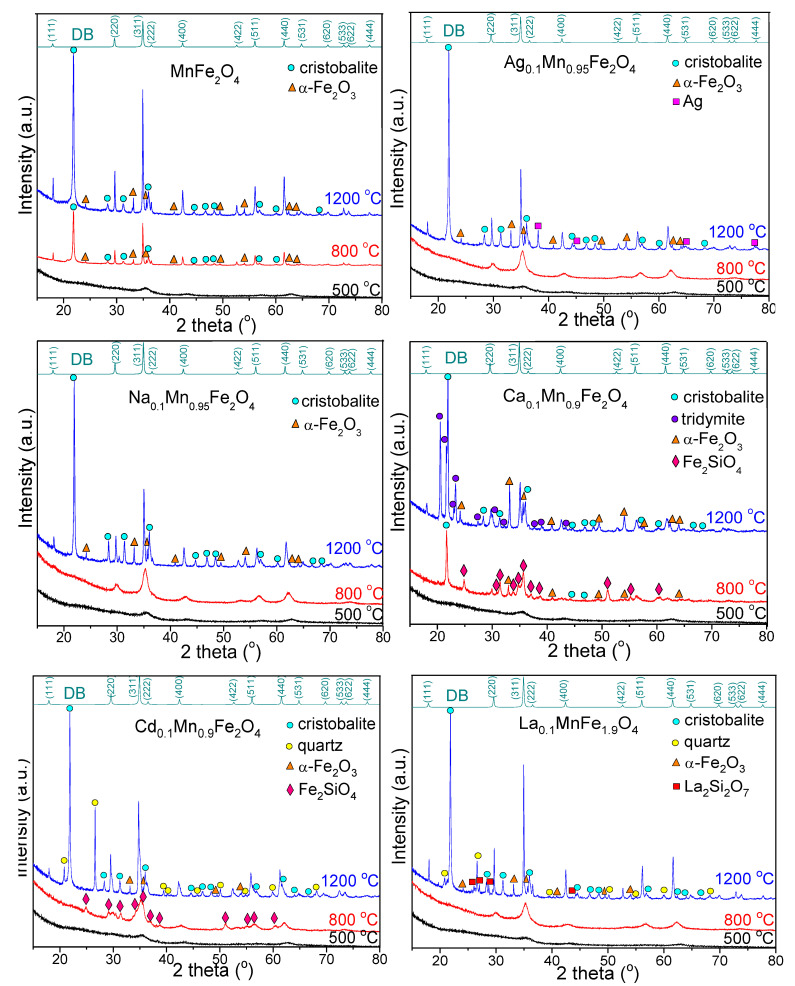
XRD patterns of MnFe_2_O_4_, Ag_0.1_Mn_0.95_Fe_2_O_4_, Na_0.1_Mn_0.95_Fe_2_O_4_, Ca_0.1_Mn_0.9_Fe_2_O_4_, Cd_0.1_Mn_0.9_Fe_2_O_4_, and La_0.1_MnFe_1.9_O_4_ gels annealed at 500, 800, and 1200 °C.

The XRD patterns of gels annealed at 500, 800 and 1200 °C are presented in Figure 2. The diffraction peaks of MnFe_2_O_4_ gels match the reflection planes of (111), (220), (311), (222), (400), (422), (511), (440), (531), (620), (533), (622) and (444) confirming the presence of pure, low–crystallized MnFe_2_O_4_ (JCPDS #00–010–0319) phase with a cubic spinel structure (space group *Fd3m*) [16]. At 500 °C, single-phase poorly crystallized MnFe_2_O_4_ is remarked, while at 800 and 1200 °C, the better crystallized MnFe_2_O_4_ is accompanied by α–Fe_2_O_3_ (JCPDS #00–033–0664) and cristobalite (JCPDS #00–074–9378) [5,16]. XRD patterns of all doped ferrites annealed at 500 °C present single-phase, low-crystallized MnFe_2_O_4_. Contrary to undoped MnFe_2_O_4_, for MnFe_2_O_4_ doped with monovalent (Ag^+^ and Na^+^) ions annealed at 800 °C, the presence of single-phase crystalline MnFe_2_O_4_ is remarked. 

For Ag_0.1_Mn_0.95_Fe_2_O_4_ annealed at 1200 °C, besides the MnFe_2_O_4_ crystalline phase, cristobalite and metallic Ag (JCPDS #00–033–0664) are also formed, indicating the presence of unreacted Ag in the SiO_2_ matrix. The diffraction patterns of MnFe_2_O_4_ doped with divalent (Ca^2+^ and Cd^2+^) ions annealed at 800 °C display various secondary phases. For Cd_0.1_Mn_0.9_Fe_2_O_4_, the MnFe_2_O_4_ crystalline phase is accompanied by Fe_2_SiO_4_ (JCPDS #00–071–1400), while for Ca_0.1_Mn_0.9_Fe_2_O_4_, the barely crystalline MnFe_2_O_4_ is attended by Fe_2_SiO_4_, α–Fe_2_O_3_, and cristobalite. At 1200 °C, the MnFe_2_O_4_ is accompanied by the crystalline phases belonging to SiO_2_ matrix (cristobalite and tridymite (JCPDS #00–074–8988)) and α–Fe_2_O_3_ for Ca_0.1_Mn_0.9_Fe_2_O_4_, and cristobalite, quartz (JCPDS #00–079–1910), and α–Fe_2_O_3_ (in a smaller amount than in the case of Ca_0.1_Mn_0.9_Fe_2_O_4_) for Cd_0.1_Mn_0.9_Fe_2_O_4_. For MnFe_2_O_4_ doped with trivalent metal ions (La_0.1_MnFe_1.9_O_4_), single-crystalline-phase MnFe_2_O_4_ at 800 °C, and additional secondary phases (cristobalite, quartz, α–Fe_2_O_3_, and La_2_Si_2_O_7_ (JCPDS #00–081–0461)) are observed at 1200 °C. Generally, the micron-sized SiO_2_ is crystalline, while the amorphous SiO_2_ refers to particle sizes up to 100 nm. Thus, in the case of MnFe_2_O_4_ doped with divalent (Ca^2+^, Cd^2+^) and trivalent (La^3+^) ions annealed at 1200 °C, three polymorph crystalline phases of the SiO_2_ matrix (cristobalite, quartz and tridymite) are formed, while in the case of doping with divalent (Ca^2+^ and Cd^2+^) ions annealed at 800 °C, the presence of crystalline Fe_2_SiO_4_ is remarked. 

The structural parameters, i.e., crystallite size (D_XRD_), degree of crystallinity (DC), lattice constant (a), unit cell volume (V), distance between magnetic ions—hopping length in A (d_A_) and B (d_B_) sites, physical density (d_p_), X-ray density (d_XRD_), and porosity (P) of gels annealed at 500, 800, and 1200 °C determined by XRD are displayed in Table 1. 

The effect of doping with various ions results in lower D_XRD_, i.e., 14.2–10.2 nm (500 °C), 16.7–14.7 nm (800 °C), and 66.3–40.1 nm (1200 °C) compared to undoped MnFe_2_O_4_. By increasing the temperature, the diffraction peaks become narrower and sharper indicating that the crystallite size increases and the surface area decreases. The D_XRD_ increases with the annealing temperature due to the crystallite agglomeration without recrystallization, leading to the formation of a single crystal instead a polycrystalline structure at high temperatures (1200 °C) [2,3,4,5]. The DC was calculated as the ratio between the area of the crystalline contribution and the total area. The lattice constant of undoped and doped MnFe_2_O_4_ gels increases with the annealing temperature, but the replacement of Mn^2+^ ion by metal ions leads to a decrease in lattice constant and the formation of a compositionally homogeneous solid solution. Moreover, the variation in the lattice constant causes internal stress and defeats further grain growth during the annealing process [2,3,4,5,16,33,35]. When a large-sized La^3+^ ion (1.6061 Å) replaces the small-sized Fe^3+^ ion (0.645 Å) initiates higher asymmetry in the lattice structure [6]. The increase in molecular weight of ferrites is more significant than the increase in volume (V, Table 1), but the molecular weight is more affected by the increase in unit cell volume [2,3,4,5]. The distance between magnetic ions (d, hopping length) in A and B sites of gels annealed at 800 and 1200 °C is higher for undoped MnFe_2_O_4_ than doped MnFe_2_O_4_ (Table 1). The lower value of physical density (d_p_, Table 1) of undoped MnFe_2_O_4_ compared to doped MnFe_2_O_4_ could be attributed to the formation of pores through the synthesis processes [2,3,4,5]. The variation in XRD and physical densities (d_XRD_ and d_p_, Table 1) caused by small fluctuations in lattice constant can be explained by considering the changes in the cation distribution within the A and B sites [2,3,4,5,16,33,35]. The rapid densification during the annealing process leads to a slight decrease in porosity (P, Table 1) with the increase in annealing temperature [2,3,4,5]. The P value of doped MnFe_2_O_4_ is lower than that of undoped MnFe_2_O_4_. The decrease in P with the increase in d_p_ may be the consequence of the different grain sizes, while by annealing, the growth of the irregular shape grains occurs [2,3,4,5]. Concluding, D_XRD_, DC, a, V, d_A_, d_B_ and d_p_ increase, while d_XRD_ and P decrease with increasing annealing temperature.

### 3.3. Morphological Analysis

**Figure 3:**
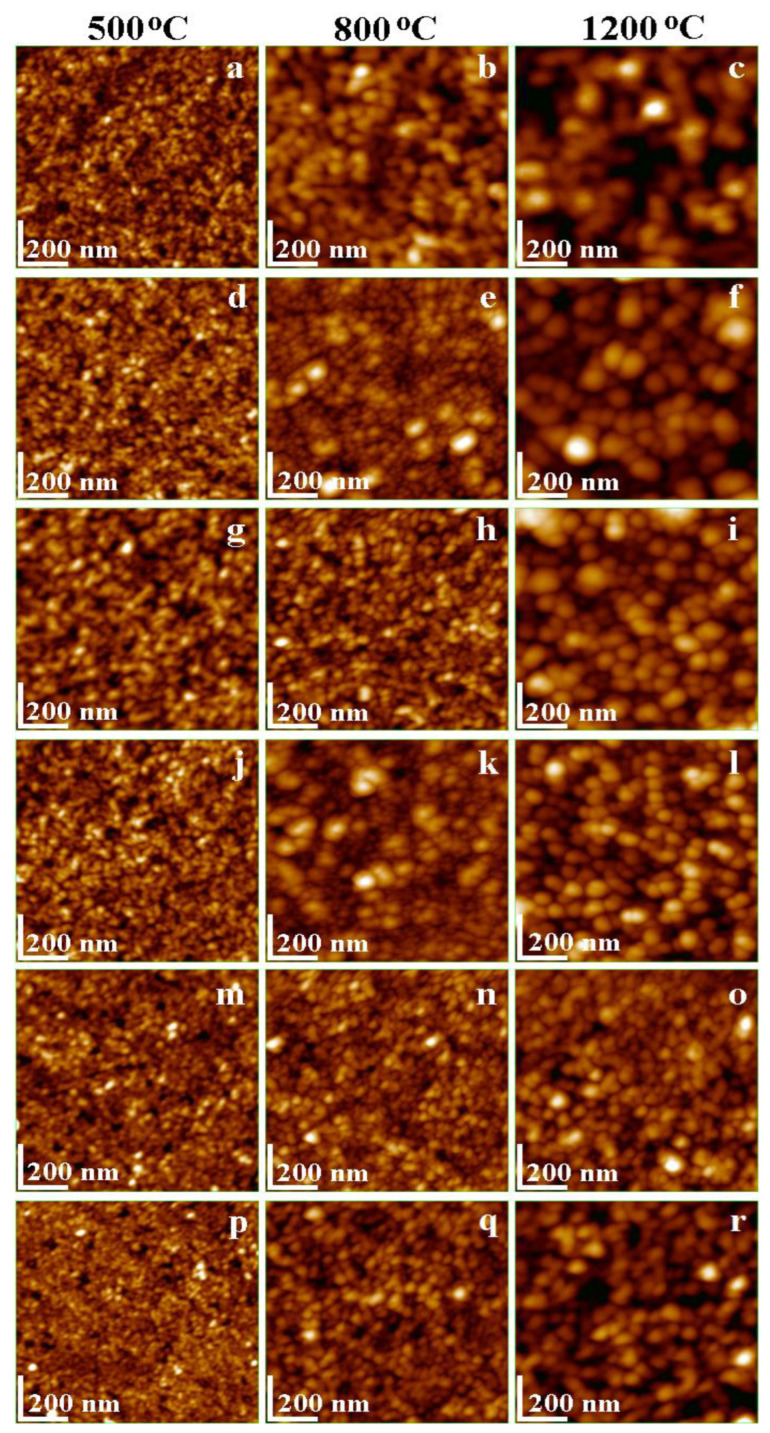
AFM topographic images of MnFe_2_O_4_ (**a**–**c**), Ag_0.1_Mn_0.95_Fe_2_O_4_ (**d**–**f**), Na_0.1_Mn_0.95_Fe_2_O_4_ (**g**–**i**), Ca_0.1_Mn_0.90_Fe_2_O_4_ (**j**–**l**), Cd_0.1_Mn_0.90_Fe_2_O_4_ (**m**–**o**), La_0.1_MnFe_1.9_O_4_ (**p**–**r**), nanoparticles annealed at 500, 800 and 1200 °C.

The sample morphology was investigated through the AFM microscopy, the nanoparticles disposal in the adsorbed thin film and their shape and size is better visible in the topographic images in Figure 3. The increase in the particle diameter with the annealing temperature evidences the evolution of the crystalline phase as a function of temperature. The diameter of pure, spherical MnFe_2_O_4_ nanoparticles (Figure 3a–c) is strongly influenced by the annealing temperature (15 nm at 500 °C, 18 nm at 800 °C and 70 nm at 1200 °C). These values are in good agreement with the crystallite size estimated by XRD data and demonstrate that the observed nanoparticles are mono–crystalline (each observed nanoparticle represents one crystallite). At low annealing temperatures, the nanoparticles are uniformly distributed onto the thin film, but at 800 °C the distribution is slightly changed due to the increase in the crystallite size domains and significantly changed at 1200 °C due to the growth of crystallite size. These results are in good agreement with previous data reported regarding MnFe_2_O_4_ nanoparticles [36]. 

At low annealing temperatures, Ag^+^ doping has a low impact on the size and shape of the nanoparticles, i.e., the particle diameter is around 13 nm (Figure 3d) at 500 °C, while at 800 °C few big nanoparticles (35–40 nm) are surrounded by a uniform and compact film of 16 nm particles (Figure 3e). However, by annealing at 1200 °C, the diameter of spherical shape particles of 57 nm has a uniform distribution in the thin film (Figure 3f). Similarly, Na^+^ doping (Figure 3g–i) does not significantly influence the particle diameter compared to that of undoped MnFe_2_O_4_. The diameter of particles at 500 °C (17 nm) and 800 °C (20 nm) and 1200 °C (45 nm) decreases compared to that of undoped MnFe_2_O_4_. The decrease in the ferrite particle size by doping with monovalent (Ag^+^, Na^+^) ions and annealing at 1200 °C is most probably due to the shrinkage of the crystal lattice and dependence of particle size on annealing temperature [37,38]. 

Ca^2+^ doping leads to the formation of spherical nanoparticles with a diameter depending on the annealing temperature. Accordingly, low temperature annealing generates fine nanoparticles (12 nm), in good agreement with the crystallite sizes of about 10 nm (Figure 3j), while by increasing the annealing temperature to 800 °C (Figure 3k), the particle diameter also increases, resulting in nanoparticles of about 40–45 nm surrounded by smaller nanoparticles (18 nm). By annealing at 1200 °C, the particle diameter increases to 60 nm (Figure 3l), slightly lower than that of undoped MnFe_2_O_4_, most probably due to some nanostructure refinement induced by Ca^2+^ doping. At high annealing temperatures, Cd^2+^ doping influences the evolution of MnFe_2_O_4_ nanoparticles, but their shape is maintained spherical. This fact agrees with the XRD data indicating that Cd^2+^ doping leads to a decrease in crystallite size. A compact, thin film of well-individualized spherical nanoparticles with an average diameter of about 14 nm are observed at 500 °C (Figure 3m) and 8 nm at 800 °C (Figure 3n), while at 1200 °C the diameter of the particles is 58 nm (Figure 3o), due to better development of the crystalline phase within the ferrite grains.

La^3+^ doping has a moderate influence on the particle diameter compared to undoped MnFe_2_O_4_. Thermal treatment at 500 and 800 °C leads to forming small spherical nanoparticles of about 13 nm, respectively 19 nm (Figure 3p,q). However, higher temperatures (1200 °C) result in nanoparticles of about 55 nm (Figure 3r), the well-contoured and individualized nanoparticles proving a solid consolidation of crystalline phase, in good agreement with the XRD data.

**Figure 4:**
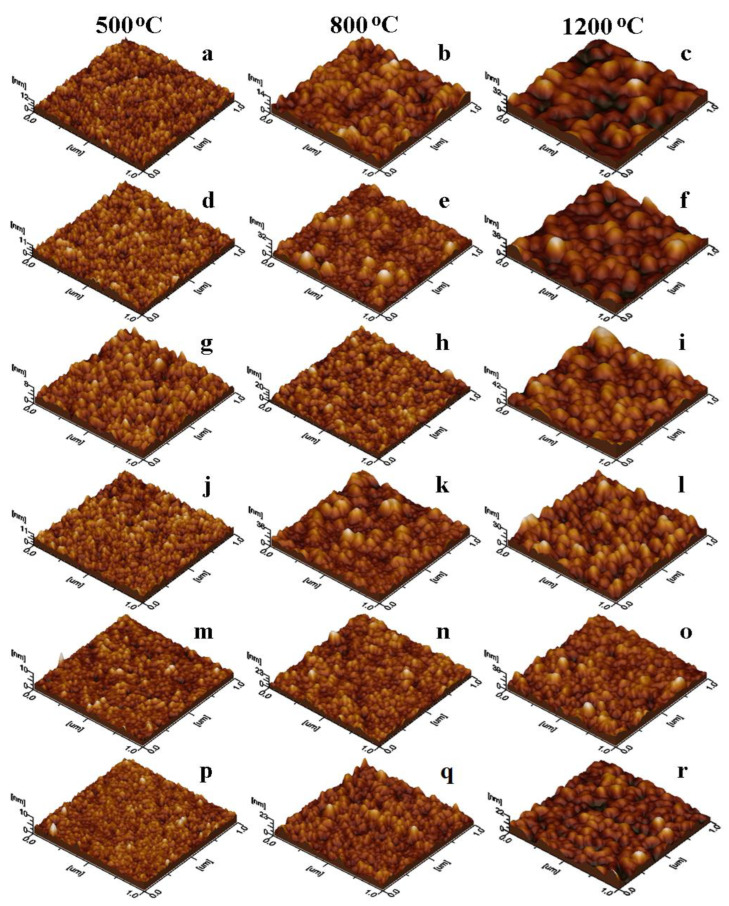
Tridimensional profiles of MnFe_2_O_4_ (**a**–**c**), Ag_0.1_Mn_0.9_Fe_2_O_4_ (**d**–**f**), Na_0.1_Mn_0.95_Fe_2_O_4_ (**g**–**i**), Ca_0.1_Mn_0.95_Fe_2_O_4_ (**j**–**l**), Cd_0.1_MnFe_1.9_O_4_ (**m**–**o**), and La_0.1_Mn_0.9_Fe_2_O_4_ (**p**–**r**) nanoparticles annealed at 500, 800, and 1200 °C.

Thin film topography is better evidenced by the tridimensional images in Figure 4, the revealed aspects being in close relation with roughness and the other surface parameters presented in Table 2. The thin film surface was measured on the AFM tridimensional profiles in Figure 4 and the values are shown in Table 2. The surface irregularities significantly increase at 1200 °C, as the surface of thin film is bigger for the gels annealed at this temperature.

All ferrite nanoparticles released into the aqueous solutions are well individualized and were adsorbed uniformly onto the glass slide forming well-structured compact thin films. Depending on the annealing temperature, different topographies were remarked. In the case of gels annealed at 500 °C, the nanoparticles form a very thin uniform film with heights of 8 to 12 nm (Figure 4a,d,g,j,m,p) and low (0.5–1.34 nm) surface roughness (Table 2). The roughest film was obtained by the undoped MnFe_2_O_4_ and the smoothest for La^3+^ doped MnFe_2_O_4_ nanoparticles. Thin films obtained by the adsorption of the Na^+^, Cd^2+^, and La^3+^-doped MnFe_2_O_4_ nanoparticles annealed at 800 °C (Figure 4h,n,q) are compact and smooth, dominated by relatively small particles uniformly distributed on the film surface (Table 2). The other gels annealed at 800 °C present a few big particles mixed between the average-sized ones, generating a relatively irregular surface (Figure 4b,e,k) with increased roughness (1.6–3.8 nm, Table 2). Bigger nanoparticles resulted after annealing at 1200 °C form a uniform, compact and well-structured thin film with relatively irregular topography (Figure 4c,f,i,l,o,r) and relatively high surface roughness (2.28–4.78 nm, Table 2). Doped MnFe_2_O_4_ thin films with similar roughness were obtained by various methods, such as sputtering [39], spin coating [40], and spray pyrolysis [41].

### 3.4. Magnetic Properties

For the ideal (containing no defects) MnFe_2_O_4_ ferrites, the magnetic properties are dictated by the antiferromagnetic interactions between the magnetic cations distributed between the A and B sites of the spinel structure. By doping with different ions, this distribution can be changed to manipulate the main magnetic parameters of the samples. The distribution of M^2+^ ions between the A and B sites in some ferrites can be modified by heat treatment, depending on whether the compounds are slowly cooling down from a high temperature or are quenched to a lower temperature [42]. The grain boundaries contain unreacted phases with isolate disordered magnetic moments for polycrystalline samples. In the case of nanoparticles, a large surface-to-volume ratio implies that many atoms are at the surface or near the surface with the associated spin distortion due to the surface effects [35,43,44]. This will make dominant the behavior of the surface atoms and from the close vicinity of the surface of the nanoparticle particle over those from the core as shown in Table 2, the annealing temperature strongly affecting the average nanoparticles diameter. The smaller the particles’ size, the more different their magnetic behavior is compared to the bulk material behavior. Thus, magnetic properties are influenced by the crystalline structure, defects, and cationic distribution, which can be controlled by both ion doping and annealing temperature [42].

**Figure 5:**
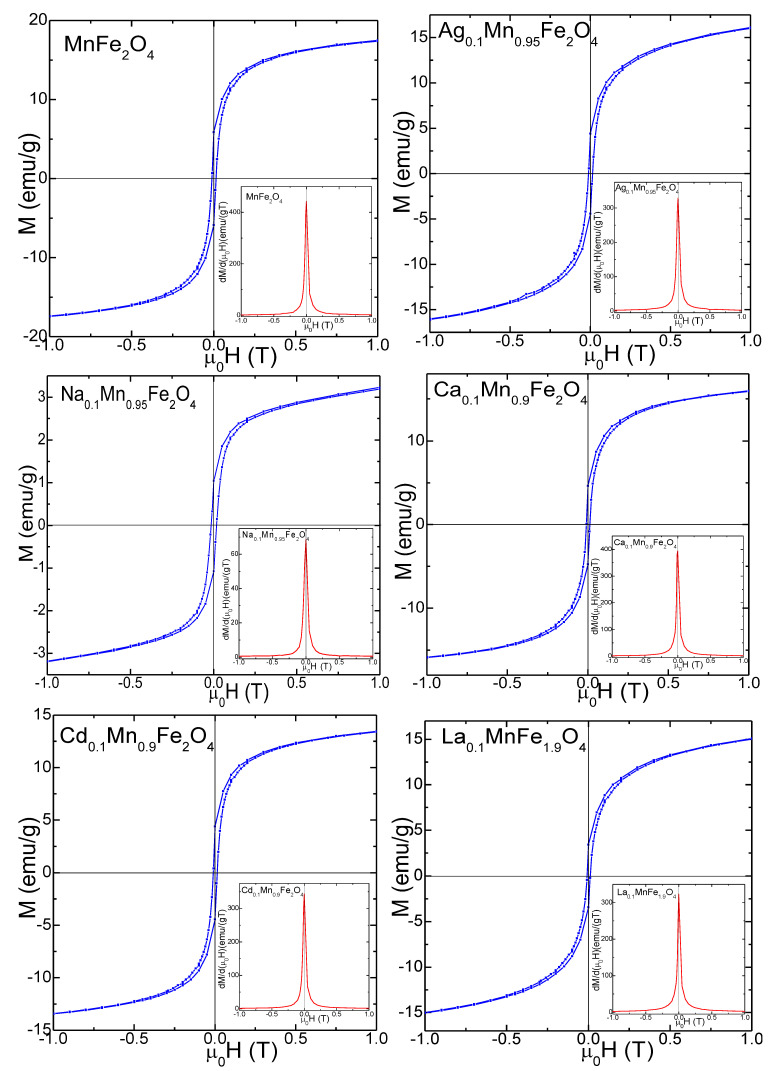
Magnetic hysteresis loops and the magnetization first derivatives of MnFe_2_O_4_, Ag_0.1_Mn_0.95_Fe_2_O_4_, Na_0.1_Mn_0.95_Fe_2_O_4_, Ca_0.1_Mn_0.9_Fe_2_O_4_, Cd_0.1_Mn_0.9_Fe_2_O_4_, and La_0.1_MnFe_1.9_O_4_ gels annealed at 800 °C.

**Figure 6:**
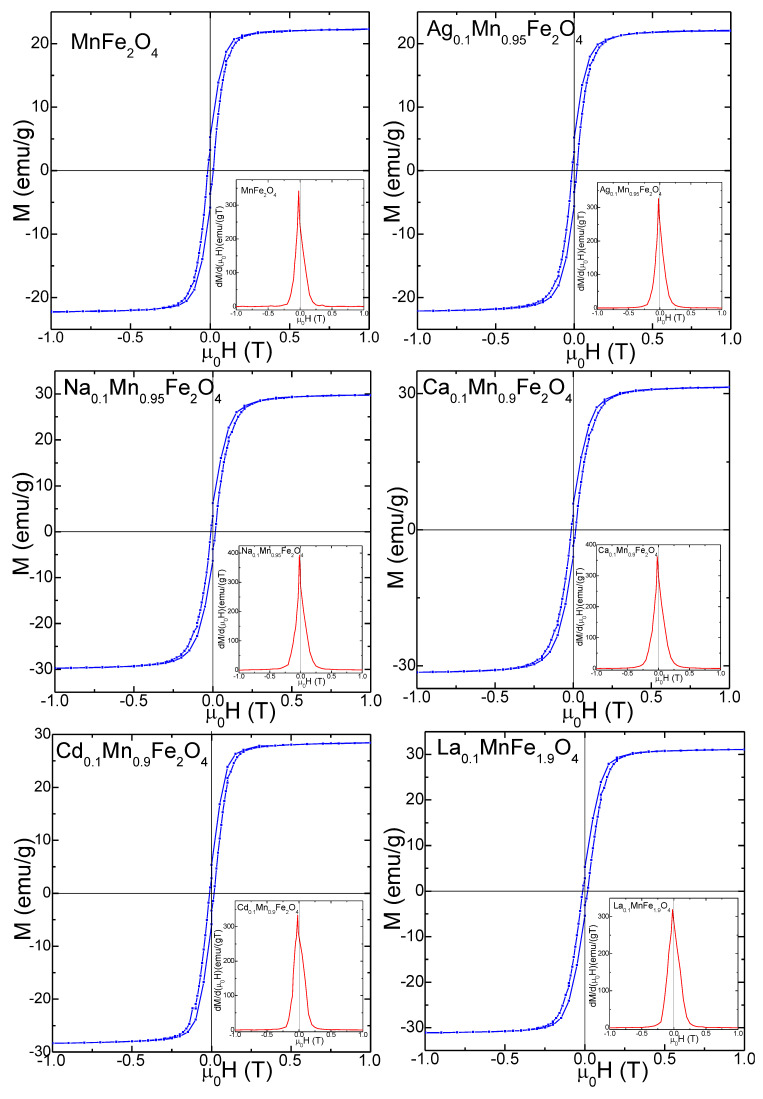
Magnetic hysteresis loops and the magnetization first derivatives MnFe_2_O_4_, Ag_0.1_Mn_0.95_Fe_2_O_4_, Na_0.1_Mn_0.95_Fe_2_O_4_, Ca_0.1_Mn_0.9_Fe_2_O_4_, Cd_0.1_Mn_0.9_Fe_2_O_4_, and La_0.1_MnFe_1.9_O_4_, gels annealed at 1200 °C.

The magnetic hysteresis loops, *M*(*µ*_0_*H*) and the first derivatives *dM/d*(*μ*_0_*H*) of gels annealed at 800 °C (Figure 5) and 1200 °C (Figure 6) indicate a ferromagnetic behavior. The single maximum in the *dM/d*(μ_0_*H*) vs. *μ*_0_*H* curves close to the coercive field indicates the occurrence of a single magnetic phase, specific for crystalline samples [5]. The magnetic hysteresis loops indicate small coercive fields (*H*_C_) ascribed to particle coalescence during annealing and to relatively low saturation magnetization (*M_S_*) values [2,3,4,5,16,33,35]. For all doping ions, the particle sizes increase with increasing annealing temperature. For the gels annealed at 1200 °C, the magnetization saturates in low magnetic fields, while for those annealed at 800 °C, the saturation is reached in higher magnetic fields, suggesting a higher magnetic disorder. Smal particle size has larger magnetic disorder at the surface, containing isolated magnetic moments or canted spin, which require a higher magnetic field for saturation.

The *M_S_* of gels annealed at 800 °C decreases from 21.5 to 6.4 emu/g for doped MnFe_2_O_4_ compared to the undoped MnFe_2_O_4_. The small-size particles have a large surface-to-volume ratio, indicating that the high surface defect density is responsible for the *M_S_* ‘depreciation [2,3,4,5]. Moreover, the magnetically dead layer at the particle surface containing broken chemical bonds, lattice defects, and isolated magnetic moments contributes to the low *M_S_* value of the nanoparticles [2,3,4,5,16,33,35]. Increasing the fraction of this layer will make its behavior dominant over the behavior of the core.

For the gels annealed at 1200 °C, the *M_S_* value increases with doping from 23.3 to 32.2 emu/g (Table 3) and with the annealing temperature owing to the increase in particle size. The small-size particles have a large surface-to-volume ratio, indicating that the high surface defect density is responsible for the *M_S_* ‘depreciation [2,3,4,5]. Moreover, the existence of magnetically dead layer at the particle surface containing broken chemical bonds, lattice defects and isolated magnetic moments contribute to the nanoparticle’s low *M_S_* [2,3,4,5,16,33,35]. Increasing the fraction of this layer will make its behavior overriding over the core. According to XRD, the annealing temperature dictates the gel’s crystallinity, while the presence of secondary phases affects the magnetic properties of the nanoparticles. Moreover, the increase in particle sizes, improvement of crystallinity, increase in spin disorder. and change in the distribution of Fe^3+^ and Mn^2+^ ions between the A and B sites will result in a higher net magnetic moment [43,44]. 

The remnant magnetization (*M_R_*) decreases for the doped MnFe_2_O_4_ gels compared to undoped MnFe_2_O_4_, from 5.9 to 1.0 emu/g (at 800 °C) and from 6.5 to 5.4 emu/g (at 1200 °C). Generally, the behavior of *M_S_* and *M_R_* can be assessed using Neel’s theory via cation distribution between the A and B sites. The Ag^+^, Na^+^, Ca^2+^, Cd^2+^ and La^3+^ ions tend to occupy the A sites, Mn^2+^ ions occupy both A and B sites, while Fe^3+^ ions can be found mainly in B sites [2,3,4,5,16,33,35,44]. The net magnetization is given by the antiparallel coupling between the magnetic moments of the A and B sites in the presence of spin disorder at the surface and/ or spin canting in the presence of secondary phases. The magnetic moments of A sites are antiparallel coupled with those from B sites and the net magnetization derives from the uncompensated magnetic moment between both sites [2,3,4,5,16,33,35,44].

Compared to undoped MnFe_2_O_4_ gel, the *H*_C_ of doped MnFe_2_O_4_ gels decreases from 116 to 100 Oe for the gels annealed at 800 °C and from 160 to 120 Oe for those annealed at 1200 °C, due to the decrease in crystallite size with a typical single magnetic domain behavior (i.e., *H*_C_ increases with increasing crystallite size). The lower *H*_C_ values also suggest a spin distortion due to the surface effects, which affect the magnetocrystalline anisotropy [35,44].

Similar to *M_S_*, the magnetocrystalline anisotropy constant (*K*) for the doped MnFe_2_O_4_ gels annealed at 800 °C decreases (from 1.57 × 10^−3^ to 0.40 × 10^−3^ erg/cm^3^), while for those annealed at 1200 °C increases (from 2.34 × 10^−3^ to 3.68 × 10^−3^ erg/cm^3^) compared to undoped MnFe_2_O_4_. A possible explanation could be the decrease in particle size which results in increased surface spin disorder and related surface effects. *K* is affected by the shape and surface anisotropy of nanoparticles [45]. Since the AFM indicated that the particle shape and area do not change substantially by doping and annealing temperature, the surface anisotropy dictates the variation in *K*.

While the properties of the obtained doped MnFe_2_O_4_ nanoparticles can be further enhanced by adjusting the dopant ions content, annealing temperature, or the SiO_2_ to ferrite ratio, this study introduces valuable information on the properties of doped MnFe_2_O_4_/SiO_2_ nanocomposites. Metal ion doping and annealing temperature can have remarkable effects on the structure, morphology, and magnetic properties of the MnFe_2_O_4_/SiO_2_ nanocomposites, which allow the control of the physical properties of these nanocomposites to make them potential candidates for various applications such as microwave and communication devices, information storage systems, ferrofluid technology, gas sensors, and medical applications for magnetic hyperthermia, magnetic resonance imaging, and photocatalytic activity.

## 4. Conclusions

The effect of doping with mono- (Ag^+^, Na^+^), di– (Ca^2+^, Cd^2+^), and trivalent (La^3+^) ions and annealing temperature on the structural, morphological, and magnetic properties of MnFe_2_O_4_ were studied. Low-crystalline MnFe_2_O_4_ at low annealing temperatures and well-crystallized MnFe_2_O_4_ at high annealing temperatures were remarked. For the gels annealed at 1200 °C, in MnFe_2_O_4_ doped with divalent and trivalent metals, three crystalline phases associated with the SiO_2_ matrix (cristobalite, quartz, and tridymite) were also observed. The crystallite size, degree of crystallinity, lattice constant, unit cell volume, hopping length, and density increased, while the porosity decreased with the annealing temperature. The crystallite size estimated by XRD was in good agreement with the particle size measured by AFM, suggesting that the observed nanoparticles contain a single ferrite crystallite. Doping slightly reduced the ferrite particle diameter depending on the doping ion radius. Uniformly self-assembling ferrite nanoparticles in thin films by adsorption from an aqueous solution may be a straightforward approach for doped ferrite nanocrystalline coatings. The saturation magnetization (*M_S_*) of doped MnFe_2_O_4_ gels annealed at 800 °C decreased compared to the undoped MnFe_2_O_4_ (from 21.5 to 6.4 emu/g), while *M*_S_ of gels annealed at 1200 °C increased for the doped MnFe_2_O_4_ gels (from 23.3 to 32.2 emu/g). Similar behavior was found for the magnetocrystalline anisotropy constant (K). The coercive field (*H_c_*) decreased by doping for the gels annealed at 800 °C (from 116 to 100 Oe) and 1200 °C (from 160 to 120 Oe). The magnetocrystalline anisotropy constant (*K*) of the doped MnFe_2_O_4_ gels was lower at 800 °C (from 1.57 × 10^−3^ to 0.40 × 10^−3^ erg/cm^3^) and higher at 1200 °C (from 2.34 × 10^−3^ to 3.68 × 10^−3^ erg/cm^3^) compared to the undoped MnFe_2_O_4_. The results obtained confirm that doping and annealing temperature play an important role in tailoring the structural, morphological, and magnetic properties of doped MnFe_2_O_4_/SiO_2_ nanocomposites making them important for catalysts and biomedical applications, such as magnetic resonance imaging, biomolecule detection, and magnetic hyperthermia. Although their use in biomedical applications is still in the beginning stage, some challenges, such as tuning size, shape, and magnetic properties of nanoparticles, exploring additional dopants, and optimizing annealing conditions, require further study. Additionally, adjusting MnFe_2_O_4_′ s surface as key performance for biomedical applications should be further explored as one of the most important challenges to obtaining ferrite nanoparticles. 

## Figures and Tables

**Table 1:** Structural parameters of MnFe_2_O_4_, Ag_0.1_Mn_0.95_Fe_2_O_4_, Na_0.1_Mn_0.95_Fe_2_O_4_, Ca_0.1_Mn_0.9_Fe_2_O_4_, Cd_0.1_Mn_0.9_Fe_2_O_4_, and La_0.1_MnFe_1.9_O_4_ gels annealed at 500, 800, and 1200 °C.

Parameter	Temp (°C)	MnFe_2_O_4_	Ag_0.1_Mn_0.95_Fe_2_O_4_	Na_0.1_Mn_0.95_Fe_2_O_4_	Ca_0.1_Mn_0.9_Fe_2_O_4_	Cd_0.1_Mn_0.9_Fe_2_O_4_	La_0.1_MnFe_1.9_O_4_	Error
D_XRD_ (nm)	500	14.2	10.2	14.0	11.8	12.0	11.1	±1.3
	800	16.7	14.7	15.9	15.3	16.3	16.2	±1.6
	1200	66.3	55.4	40.1	58.0	56.5	50.0	±5.5
DC (%)	500	61.5	48.5	48.9	61.2	60.8	59.9	±5.0
	800	70.2	62.0	69.4	66.9	63.9	68.0	±6.6
	1200	90.1	88.8	86.3	85.5	89.5	88.6	±8.7
a (Å)	500	8.445	8.414	8.427	8.409	8.418	8.441	±0.01
	800	8.485	8.457	8.462	8.443	8.467	8.478	±0.01
	1200	8.544	8.504	8.510	8.491	8.533	8.517	±0.01
V (Å^3^)	500	602.3	595.7	598.4	594.6	596.5	601.4	±0.01
	800	610.9	604.6	605.9	601.9	607.0	609.4	±0.01
	1200	623.7	615.0	616.3	612.2	621.3	617.8	±0.01
d_A_ (Å)	500	3.657	3.643	3.649	3.641	3.645	3.655	±0.01
	800	3.674	3.662	3.664	3.656	3.666	3.671	±0.01
	1200	3.700	3.682	3.685	3.677	3.695	3.688	±0.01
d_B_ (Å)	500	2.986	2.975	2.979	2.973	2.976	2.984	±0.01
	800	2.999	2.990	2.992	2.985	2.994	2.997	±0.01
	1200	3.021	3.007	3.009	3.002	3.017	3.011	±0.01
d_p_ (g/cm^3^)	500	4.133	4.375	4.187	4.298	4.340	4.388	±0.01
	800	4.255	4.554	4.299	4.420	4.471	4.472	±0.01
	1200	4.334	4.633	4.474	4.549	4.555	4.577	±0.01
d_XRD_ (g/cm^3^)	500	5.087	5.322	5.110	5.119	5.264	5.278	±0.01
	800	5.015	5.244	5.047	5.057	5.173	5.209	±0.01
	1200	4.912	5.155	4.962	4.972	5.054	5.138	±0.01
P (%)	500	18.7	17.8	18.0	16.0	17.5	16.9	± 1.6
	800	15.1	13.2	14.8	12.6	13.6	14.1	± 1.2
	1200	11.8	10.1	9.83	8.51	9.87	10.9	± 1.0

**Table 2:** Average nanoparticles diameter (D_AFM_), thin film height (H), roughness (Rq) and surface of MnFe_2_O_4_, Ag_0.1_Mn_0.95_Fe_2_O_4_, Na_0.1_Mn_0.95_Fe_2_O_4_, Ca_0.1_Mn_0.90_Fe_2_O_4_, Cd_0.1_Mn_0.9_Fe_2_O_4_, and La_0.1_Mn_1_Fe_1.9_O_4_ gels annealed at 500, 800, and 1200 °C.

Gel	Temperature (°C)	H (nm)	Rq (nm)	D_AFM_ (nm)	Surface (nm^2^)
MnFe_2_O_4_	500	12	1.34	15	1012
	800	14	1.60	18	1017
	1200	32	3.73	70	1022
Ag_0.1_Mn_0.95_Fe_2_O_4_	500	11	1.28	13	1019
	800	32	3.08	16	1023
	1200	38	4.78	57	1035
Na_0.1_Mn_0.95_Fe_2_O_4_	500	8	0.92	17	1014
	800	20	2.29	20	1031
	1200	42	5.25	45	1044
Ca_0.1_Mn_0.9_Fe_2_O_4_	500	11	1.29	12	1017
	800	36	3.81	18	1027
	1200	30	4.17	60	1039
Cd_0.1_Mn_0.9_Fe_2_O_4_	500	10	0.50	14	1009
	800	23	2.41	18	1037
	1200	39	3.87	58	1054
La_0.1_MnFe_1.9_O_4_	500	10	0.54	13	1014
	800	23	2.40	19	1030
	1200	22	2.28	55	1022
Error	–	± 1.0	± 0.20	± 5.0	±5.0

**Table 3:** Magnetic parameters of MnFe_2_O_4_, Ag_0.1_Mn_0.95_Fe_2_O_4_, Na_0.1_Mn_0.95_Fe_2_O_4_, Ca_0.1_Mn_0.9_Fe_2_O_4_, Cd_0.1_Mn_0.9_Fe_2_O_4_, and La_0.1_MnFe_1.9_O_4_ gels annealed at 800 and 1200 °C.

Parameter	Temp (°C)	MnFe_2_O_4_	Ag_0_._1_Mn_0.95_Fe_2_O_4_	Na_0_._1_Mn_0.95_Fe_2_O_4_	Ca_0.1_Mn_0.9_Fe_2_O_4_	Cd_0_._1_Mn_0.9_Fe_2_O_4_	La_0_._1_MnFe_1.9_O_4_	Errors
*M_s_* (emu/g)	800	21.5	21.0	6.4	19.5	17.5	20.1	±1.1
	1200	23.3	26.3	30.7	32.2	29.9	31.7	±2.8
*M_R_* (emu/g)	800	5.9	4.4	1.0	4.6	4.4	3.4	±0.4
	1200	6.5	5.4	6.2	5.8	5.5	5.6	±0.6
*H_c_* (Oe)	800	116	112	100	105	110	113	±10
	1200	160	145	120	125	140	156	±15
*K*·10^3^ (erg/cm^3^)	800	1.57	1.48	0.40	1.29	1.21	1.43	±0.10
	1200	2.34	2.39	3.68	2.53	2.63	3.11	±0.25

## Data Availability

The data that support the findings of this study are available on request from the corresponding author.
